# Lithocholic acid attenuates DON-induced inflammatory responses via epigenetic regulation of DUSP5 and TRAF5 in porcine intestinal epithelial cells

**DOI:** 10.3389/fvets.2025.1493496

**Published:** 2025-02-28

**Authors:** Shiqi Wang, Xiaoxu Peng, Qi Zhu, Sichen Lu, Ping Hu, In Ho Kim, Hao-Yu Liu, Wael Ennab, Madesh Muniyappan, Demin Cai

**Affiliations:** ^1^College of Animal Science and Technology, Yangzhou University, Yangzhou, China; ^2^Department of Animal Resource and Science, Dankook University, Cheonan, Choongnam, Republic of Korea; ^3^Jiangsu Key Laboratory of Animal Genetic Breeding and Molecular Design, College of Animal Science and Technology, Yangzhou University, Yangzhou, China

**Keywords:** epigenetics, histone modification, porcine, deoxynivalenol, lithocholic acid

## Abstract

Deoxynivalenol (DON) is the most common mycotoxin that frequently contaminates human food and animal feed, resulting in intestinal diseases and systemic immunosuppression. Lithocholic acid (LCA) exhibits various pharmacological activities. RNA-seq and ChIP-qPCR analysis were used in the current study to investigate the protective mechanism of LCA for DON-induced inflammatory Responses via Epigenetic Regulation of DUSP5 and TRAF5 in porcine ileal epithelial cell lines (IPI-2I) cells. The IPI-2I cells were treated with the vehicle group, 250 ng/mL DON, 20 μmol/L LCA, 250 ng/mL DON+ 20 μmol/L LCA for 24 h could induce inflammatory Responses via Epigenetic Regulation of DUSP5 and TRAF5 in IPI-2I cells. By analyzing the transcriptional profiles of DON and LCA-treated IPI-2I, we observed significant transcriptional changes in IPI-2I cells. Further analysis of up-and down-regulated differential genes revealed the enrichment of pathways closely related to inflammation and apoptosis, such as the MAPK signaling pathway, IL17 signaling pathway, and Wnt signaling pathway. An upregulated (*p* < 0.05) relative mRNA expression level of RAP1B, GDNF, FGF2, IL1R1, RAPGEF2, DUSP5, TGFB3, CACNA1G, TEK and RPS6KA2 were noted in IPI-2I exposed to DON. DON-exposed IPI-2I cells dramatically enhanced (*p* < 0.05) histone marks associated with transcriptional activation, H3K9ac, H3K18ac, H3K27ac, H3K4me1, H3K9bhb, H3K18bhb Pol-II and Ser5 Pol-II at the enhancers of DUSP5 and TRAF5. Overall, our findings provide a theoretical basis for understanding the mechanism of action of LCA in attenuating DON-induced intestinal injury and for better understanding the potential of LCA as a treatment or prevention of mycotoxin-associated intestinal diseases in swine production.

## 1 Introduction

One of the most prevalent mycotoxins is deoxynivalenol (DON), also known as vomitoxin. Fusarium graminearum and Fusarium culmorum are the primary producers of DON, which contaminates human and animal food (1). It is a low molecular weight secondary metabolite produced by Fusarium (2). One of the most common mycotoxins in crops such as corn and wheat, DON is chemically stable (3). It poses a serious risk to the ecosystem due to its wide dispersion and chemical persistence, and through the food chain, it may ultimately affect human and animal health (4). According to recent research, DON’s average global detection rate is as high as 59%, with the highest detection rates in Europe and Asia (5). Vomiting, loss of appetite, growth retardation, intestinal bleeding, disorders of the immune system, diarrhea, abdominal discomfort and even death can result from the ingestion of DON (6). Besides, DON can seriously affect gut health (4), inhibiting the synthesis of tight junction proteins in intestinal epithelial cells and reducing intestinal resistance to harmful microbes (7). Furthermore, chronic intestinal exposure to DON can affect intestinal barrier function (8). On the one hand, DON can prevent intestinal epithelial cells from synthesizing proteins, break down tight junction protein structures, disrupt the intestinal barrier, increase intestinal epithelium permeability, reduce nutrient absorption and transport efficiency, and induce nuclear factor-κB (NF-κB) and mitogen-activated protein kinase (MAPK) signaling pathways in intestinal cells. This results in overexpression of immune and inflammatory factors, release of chemokines, and ultimately apoptosis (2, 9, 10). A previous study in a swine jejunal epithelial cell line found elevated proinflammatory cytokine mRNA levels in response to physiologically relevant doses of DON (11). Determining the adverse effects of the mechanism of DON is essential to developing new approaches to prevent and treat diseases caused by DON (12). DON activates caspase-12, which reduces intestinal barrier integrity in weaned pigs (13). Studies conducted *in vitro* have demonstrated that at the cellular level, it downregulates the tight junction protein claudin-4 expression in intestinal epithelial cells, induces inflammatory responses, and induces apoptosis (14). Therefore, maintaining intestinal barrier integrity, immune balance, and gut microbial homeostasis could be an attractive way to reduce DON-induced toxicity in pigs (15).

Bile acids have diverse roles in physiological responses, including regulating cholesterol metabolism, the bile acid cycle, and absorption of fat-soluble nutrients (16, 17). Transport proteins in the membranes of hepatocytes and ileal epithelium allow bile acids to enter the cells (18); these proteins regulate the physiological signaling of nuclear receptors (NRs) and are essential for the immune system, inflammation, and oxidative stress (19). Lithocholic acid (LCA), commonly referred to as 3α-hydroxy-5β-solan-24-oic acid, is a monohydroxy bile acid produced by intestinal bacteria from chenodeoxycholic acid or ursodeoxycholic acid (20, 21). Although LCA is thought to be harmful to hepatocytes, it acts as a detergent to absorb fat in the intestine (22). In particular, LCA is associated with carcinogens. High levels of LCA (50 mg) can cause oxidative stress, damage DNA, and accelerate tumor growth by preventing DNA repair enzymes from working and promoting cell division (23, 24). Recent studies have shown that LCA may play a role in managing liver and intestinal inflammation (25, 26), as well as exhibiting anti-aging and anti-tumor properties (27), as well as antibacterial effects (28). Previous investigations have demonstrated that LCA and its derivatives exhibit anti-inflammatory and tumor-suppressing properties (29). For instance, hepatocellular carcinoma cells are selectively induced to undergo cell death upon exposure to galactosylated poly(ethylene glycol)-lithocholic acid, while normal hepatocytes remain healthy (30). In addition to maintaining the intestinal flora and lowering the risk of intestinal illnesses, it functions as a vitamin D receptor ligand to help shield the gut from outside damage (31, 32). Our previous research has shown that LCA could protect lipid metabolism to reduce DON-induced IPI-2I cytotoxic apoptosis (33). PPARγ-mediated epigenetic transcriptional reprogramming, involving histone acetylation and emulsification, has been shown to protect intestinal epithelial cells against DON-induced oxidative stress and inflammatory damage (3). Several PPARγ and other nuclear receptor coactivators and corepressors are known to possess inherent histone-modifying properties (10). The basic building block of chromatin structure, a nucleosome is made up of DNA wrapped around the octameric core and two of each of the core histones, H2A, H2B, H3, and H4. With their N-terminal tails extending from the nucleosome, the H3 and H4 histones are especially vulnerable to post-translational changes by certain enzymes. Histone acetylation, methylation, and phosphorylation are examples of chromatin modifications that constitute significant epigenetic regulatory mechanisms (34).

In a multicellular eukaryotic organism, the entire genome is usually housed in the nucleus of each cell and organized into a highly complex structure called chromatin. The nucleosome comprises eight core histones (H2A, H2B, H3, and H4) around which DNA coils in almost two turns, and it is the basic unit of chromatin. Highly conserved peptide components of histones called histone tails are generally unstructured and extend from the central nucleosome body; they undergo various post-translational modifications. Histone acetylation is a common histone post-translational modification. This modification, associated with permissive, transcriptionally active chromatin, occurs mainly in the epsilon amino groups of lysine residues in the N-terminal tails of histones H3 and H4 (35). Histone lysine methylations are post-translational modifications that have been well studied, but the functional context is more complex than that of acetylation. For example, trimethylation of histone H3 at lysine 4 (H3K4me3 and me1) is strongly linked to active genes (36). Without altering the DNA sequence, the epigenetic process can produce heritable phenotypic changes by activating or inhibiting gene expression, which ultimately results in disease. In multicellular organisms, epigenetic markers formed during development can be passed on to offspring, meaning that the same genotype can result in distinct phenotypes (37). Because epigenetic mechanisms have the power to control gene expression, they are important for the stability and function of the genome (38). Dual-specificity phosphatase 5 (DUSP5), also known as VH1-like phosphatase-3 (VH3), is a MAPK pathway (39). It has been demonstrated that mRNA and DNA methylation may alter the DUSP5 gene. Regarding mRNA modification, silencing YTHDF1, which is highly expressed in the m6A family, suppresses the expression of the DUSP5 gene, which reduces drug resistance and increases the efficacy of cancer therapy (40). In contrast, DUSP5 gene expression was negatively correlated with the level of DNA methylation (40, 41). Further investigation found that DUSP5 gene knockdown inhibited the production and secretion of pro-inflammatory factors such as TNF-α, IL-6, and IL-1β. Meanwhile, reduced DUSP5 gene expression suppressed BCG-mediated inflammatory responses in macrophages by inhibiting NF-κB pathway phosphorylation (42). Tumor necrosis factor receptor 5 (TRAF5) proteins remain part of a family of proteins involved in signal transduction from receptors of the tumor necrosis factor receptor superfamily and Toll-like receptor (TLR)/interleukin-1 (IL-1) receptor superfamily, but also by unconventional receptors for cytokines including IL-6 (43). TRAF5 was identified as a possible signaling regulator for CD40 (43) and the lymphotoxin-β receptor (44). TRAF5 is widely expressed in resting B- and T- lymphocytes (45) and is significantly expressed in spleen, thymus, and lung (46). The polymorphic TRAF5 gene regulates TRAF5 production and the release of downstream inflammatory cytokines, including TNF-α and IL-6 (47). It has been demonstrated that TRAF5 deficiency causes DDS-induced colitis; through RUNX1, TRAF5 regulates the immune response and controls the differentiation of Th1 and Th17 cells, contributing to the pathophysiology of colitis (48). TRAF5 and DUSP5 are the most promising genes to target in the resulting inflammatory diseases. Target genes can be induced to express rhythmically through dynamic histone modifications, which can alter the chromatin structure of genes and affect access to clock regulators and/or RNA polymerase II (Pol-II) (49).

This study aims to develop a viable IPI-2I cell model to study the MAPK and IL-17 signaling pathways. Furthermore, it seeks to demonstrate the impact of DON on both paths and histone modifications in the DUSP5 and TRAF5 genes.

## 2 Materials and methods

### 2.1 DON production and analysis

Fusarium graminearum strain W3008 was provided by the College of Animal Science and Technology, Yangzhou University, China. The strain was grown on potato dextrose agar at 28°C for seven days to obtain mature spores. Three hundred grams of maize, fifty grams of rice, and 140 mL of sterilized distilled water were added to a 1-liter conical flask and then autoclaved at 121°C for 20 min. Each flask was inoculated with F. graminearum at 1 × 10^6^ spores/g and incubated at 28°C and 85% humidity for 28 days. Finally, the mold-contaminated sample in each flask was dried in an air oven at 65°C overnight, mixed, and sampled to determine the DON content. The resulting coated product was confirmed to contain approximately 300 mg/kg DON. DON content was determined using the Agra Quant^®^ DON ELISA test kit following the manufacturer’s protocol (Romer Labs, Singapore).

### 2.2 Cell culture

Porcine intestinal epithelial cells, IPI-2I, were cultured in RPMI-1640 (Hyclone, UT) and supplemented with 10% fetal bovine serum (Hyclone, UT), 100 U/mL penicillin (Solarbio, Beijing, China), and 100 μg/mL streptomycin (Solarbio, Beijing, China). The cells were maintained in a 5% CO_2_, 37°C incubator. When the cells reached 90% confluency, they were digested with trypsin for 2 min and transferred to a 6-well plate after centrifugation at 1000 rpm for 5 min. When the cells reached 50% confluency, LCA (10 and 20 μmol/L) (Shanghai yuanye, Shanghai, China) stock solution dissolved in dimethyl sulfoxide (DMSO) was added to the cells and pretreated for 24 h, followed by the addition of DON (250 ng/mL) (J&K Scientific, Beijing, China) for 48 h of treatment. Finally, the cells were harvested.

### 2.3 Treatments

When the confluence of cells reached 80–90%, cells were seeded into 6 well plates and cultured for 24 h before different treatments. Based on the previous research in our laboratory (3, 33), we divided into four groups, respectively. We named it as follows: Vehicle group, DON (250 ng/mL) group, LCA (20 μmol/L) group, and DON + LCA (250 ng/mL+ 20 μmol/L) group. Different doses of LCA (Shanghai Yuanye, Shanghai, China) were added to the indicated wells for 24 h. Then, DON (J&K Scientific, Beijing, China) or DMSO was added for another 48 h.

### 2.4 RNA extraction

IPI-2I cells (5 × 10^5^ cells/well) were seeded in 6 well plates and cultured for 24 h. Following four treatments (vehicle group, 250 ng/mL DON, 20 μmol/L LCA, 250 ng/mL DON+ 20 μmol/L LCA) for 24 h, the cells were washed twice with PBS and then harvested. Total RNA was isolated using Trizol (Invitrogen, Waltham, MA, USA) according to the manufacturer’s instructions and stored at −80°C. The quantity and purity of the extracted RNA were assessed via a protein-nucleic acid analysis instrument (ND-2000UV, Thermo Fisher, Waltham, USA) and confirmed through 1% agarose gel electrophoresis. Subsequently, the RNA was converted into cDNA using the transcript All-in-One First-Strand cDNA Synthesis Super MIX for qPCR (QIAGEN, Frankfurt, Germany). The reverse transcription mixture consisted of 0.5 μg of total RNA, 5 μL of 5 × TransScript All-in-one SuperMix for qPCR, 0.5 μL of gDNA Remover, and nuclease-free H2O was adjusted to a total volume of 10 μL. The reverse transcription was carried out at 42°C for 15 min, followed by 85°C for 5 s. Post-transcription, 90 μL of nuclease-free H2O was added to the mixture, then stored at −20°C. Real-time PCR was performed using a LightCycler^®^ 480 IIReal-time PCR Instrument (Roche, Basel, Switzerland) with a PCR efficiency ranging from 94 to 105%. The PCR reaction mixture (10 μL) included 1 μL of cDNA, 5 μL of 2 × PerfectStartTM Green qPCR SuperMix, 0.2 μL of forward primer, 0.2 μL of reverse primer, and 3.6 μL of nuclease-free water. The reactions were conducted in 384-well optical plates (Roche, Basel, Switzerland) with an initial denaturation at 94°C for 30 s, followed by 45 cycles of 94°C for 5 s and 60°C for 30 s. A melting curve analysis was performed post-PCR to ensure the specificity of the PCR product. Each sample was analyzed in triplicate. Additionally, qRT-PCR was conducted using an ABI StepOne Plus Real-Time PCR System (Applied Biosystems, CA, USA) with AceQ^®^ qPCR SYBR Green Master Mix (Vazyme, Nanjing, China). The mRNA expression levels were normalized to glyceraldehyde-3-phosphate dehydrogenase (GAPDH) and quantified using the 2^−ΔΔCt^ method.

### 2.5 Data analysis of RNA sequencing

Raw reads were quality assessed and cleaned by trimming the 3′ adapter sequence using CutAdapt (50). Raw reads with an average sequencing accuracy of less than 99.9% were removed. Clean reads were aligned to porcine genome assembly *Sus scrofa* 11.1 using HISAT2 with default parameters (51). Mapping results of different genomic regions were evaluated. Read counts were calculated for each gene using HTSeq with the union strategy (52). To exclude the effects of sequencing coverage and genome length, read counts were normalized to fragments per kilobase of exon sample per million mapped fragments (FPKM), which were used as input for the following analysis. The Pearson correlation coefficient was calculated using the expression values of all genes detected between different samples. Furthermore, principal component (PC) analysis was performed among all samples using all detected genes. Differential expression analysis was performed in DESeq2 (Bioconductor version 1.6.2), and genes in the two groups with an absolute value of *p* < 0.05 and log2 (fold change) > 1 were considered differentially expressed genes (DEGs).

### 2.6 GO functional annotation and KEGG pathway enrichment analysis

The gene set enrichment analysis (GSEA 4.1.0) software was used to identify the enriched pathway profiles. In addition, statistically enriched biological processes or pathways in differentially expressed genes (DEGs) of the GO and KEGG pathways were ranked and categorized through the Metascape database^1^ and DAVID.^2^ GSEA enrichment analysis plots, KEGG enrichment bubble plots, volcano plots, and GO-pathway enrichment result circle plots were plotted through the online platform for data analysis and visualization.^3^ Gene set enrichment analysis (GSEA) was conducted to analyze the data in fragments per kilobase million (fpm), and the differential gene expression between the MLT and Vehicle groups was analyzed using the R programming language. Additionally, correlation analysis was conducted using the online protein interaction network platform (STRING)^4^ to examine gene interaction networks.

### 2.7 Venn diagram

Total RNA was extracted from IPI-2I cells treated with vehicle, 20 μmol/L LCA, DON (250 ng/mL), and DON (250 ng/mL) plus LCA (10, 20 μmol/L), respectively. Visualizations such as Venn diagrams and other types were performed on the online platform (see text footnote 3) and TBtools-II v2.011.

### 2.8 Real-time quantitative PCR

IPI-2I cells (5 × 105 cells/well) were seeded in 6 well plates and culture for 48 h. Following four treatments (vehicle group, 250 ng/mL, DON, 20 μmol/L LCA, 250 ng/mL+ 20 μmol/L DON + LCA) for 6 h, the cells were washed twice with PBS and then harvested. Total RNA was isolated using Trizol (Invitrogen, Waltham, MA, USA) according to the manufacturer’s instructions and stored at −80°C. Subsequently, the RNA was reverse-transcribed into cDNA according to the instructions (Vazyme, Nanjing, China). The mRNA expression was determined according to the instructions (Vazyme, Nanjing, China), and its relative expression was calculated using the 2^−ΔΔCT^ method.

### 2.9 ChIP-qPCR analysis

IPI-2I cells were treated with a 1% formaldehyde solution and incubated on a shaker for 12 min, followed by incubation with glycine for 10 min. After the supernatant was discarded, the cells were washed twice with PBS. Next, 3 mL of PBS was added to the culture dish, and the cells on the dish were scraped off using a cell brush. The cell suspension was then centrifuged at 2000 rpm for 5 min at 4°C. After the supernatant was discarded, the cells were resuspended in lysis buffer (1 mmol/L ethylenediaminetetraacetic acid (EDTA), 50 mmol/LN-(2-hydroxyethyl)piperazine-N′-ethanesulfonic acid (HEPES) pH 8.0, 0.5% Nonidet P-40, 140 mmol/L NaCl, 0.25% Triton X-100, 10% glycerol). The supernatant was discarded after another round of centrifugation at 2000 rpm for 5 min at 4°C. Then the cells were resuspended in wash buffer (1 mmol/L EDTA, 0.5 mmol/L ethylene glycol-bis (β-aminoethyl ether)-N, N, N′, N′-tetraacetic acid (EGTA), 10 mmol/L Tris pH 8.0, 200 mmol/L NaCl) and then again centrifuged at 2000 rpm for 5 min at 4°C. The supernatant was discarded, and the cells were resuspended in a shearing buffer (0.1% sodium dodecyl sulfate (SDS), 10 mmol/L Tris HCl pH 8.0, 1 mmol/L EDTA pH 8.0). Then, the cells were sonicated and centrifuged at 12,000 rpm for 10 min. The supernatant was incubated with magnetic beads, which were coupled with H3K9ac, H3K18ac, H3K27ac, H3K4me1 and me3, H3K9bhb, H3K18bhb, RNAPII, and RNAPII-S5P antibodies. The immune complexes were washed with LiCl wash buffer (500 mmol/L LiCl, 1% Nonidet P-40, 0.5% sodium deoxycholate, 100 mmol/L Tris pH 7.5). Proteinase K and RNase A were added for DNA extraction for the ChIP-qPCR assays.

### 2.10 Statistical analysis

GraphPad Prism 9 was used to analyze all data and mean ± SD was used to show results. At least three times in each experiment. It was considered statistically significant when *p* < 0.05.

## 3 Results

### 3.1 Analysis of pathway enrichment

**Figure 1:**
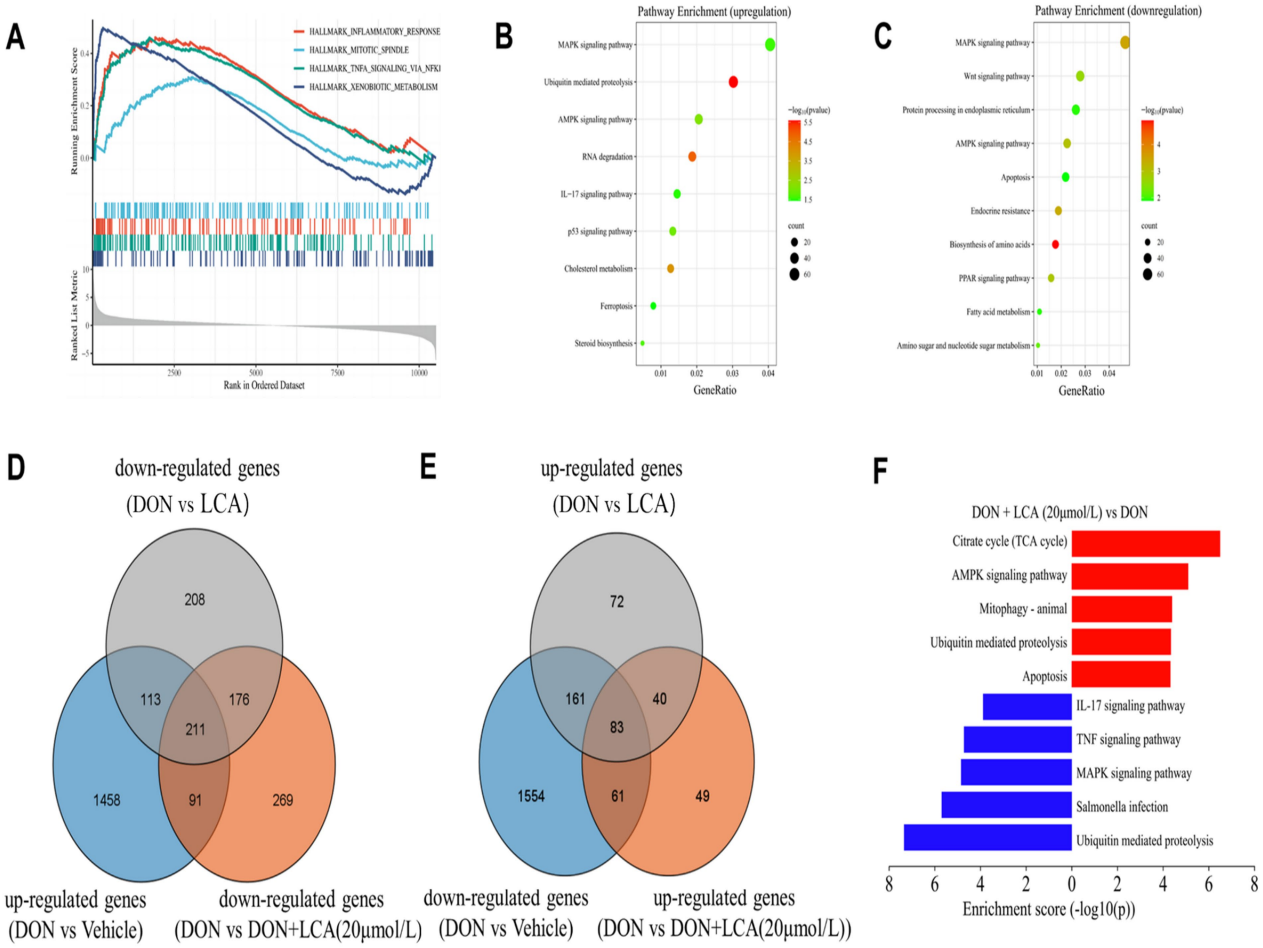
The collection of visualization graphs was obtained after comparing the sequencing results of different treatment groups. **(A)** GSEA results revealed several significant pathways enriched by DEGs between the DON group and the Vehicle group. **(B,C)** Based on the enrichment analysis of the KEGG database, the various pathways enriched by the up-regulated or down-regulated DEGs between the DON group and the vehicle group are shown separately. **(D)** By taking the intersection of genes that are significantly upregulated in the DON group compared to the vehicle group, genes that are significantly downregulated in the DON + LCA (20 μmol/L) group compared to the DON group, and genes that are significantly downregulated in the DON + LCA group compared to the DON group, to illustrate the impact of different LCA dosages on gene transcription under DON exposure. **(E)** The commonly enriched pathways by the DEGs in both the DON + LCA (20 μmol/L) group and the DON + LCA group (compared to the DON group). **(F)** Differences in activity scores of indicated pathway gene expressions using gene ontology analysis between the DON + groups and the DON + LCA (20 μmol/L) treated IPI cells. The *p*-value was the result of the DON + LCA (20 μmol/L) group.

**Figure 2:**
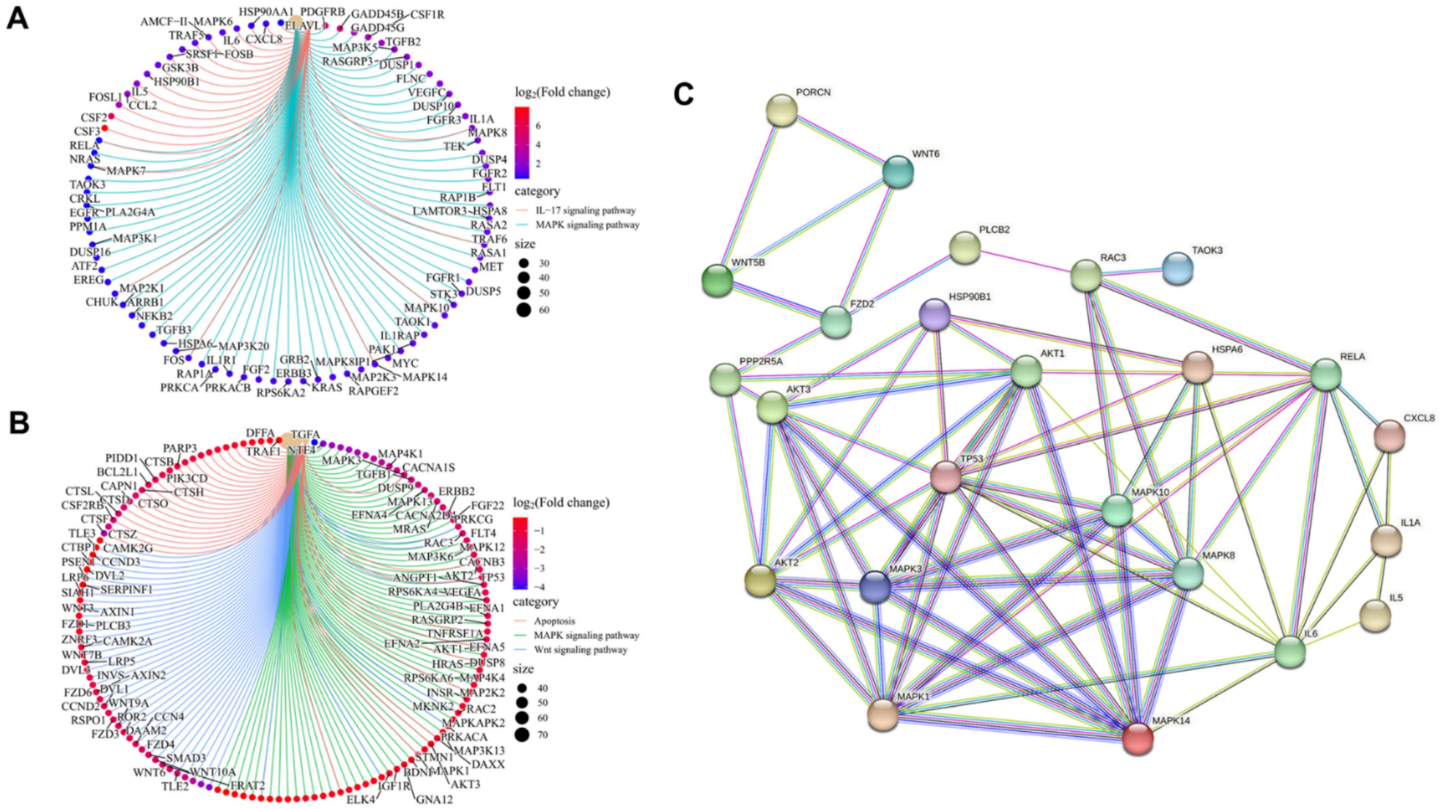
The gene transcription in the enriched pathway was specifically studied, and the relationship between genes was analyzed briefly. **(A)** Based on the up-regulated DEGs in the DON group compared to the Vehicle group, the overlapping genes between the MAPK signaling pathway and the IL-17 signaling pathway are revealed. **(B)** Based on the down-regulated DEGs in the DON group compared to the vehicle group, the overlapping genes among the MAPK signaling pathway, wnt signaling pathway, and apoptosis are revealed. **(C)** The interaction between key genes involved in the inflammatory response, MAPK signaling pathway, IL17 signaling pathway, WNT signaling pathway, and apoptosis was analyzed by STRING.

To identify key transcriptional pathways regulated by LCA + DON, transcriptome analysis was performed using IPI-2I cells from the LCA, DON, LCA + DON, and vehicle groups. We calculated the fold change of FPKM, excluded zero or nonsensical values, and then enriched the remaining dataset for enrichment analysis using GSEA version 4.1.0. The genes in the vehicle/LCA, DON, LCA + DON group were highly enriched in the inflammatory response pathway, mitotic spindle, TNF-α signaling via NF-κB and xenobiotic metabolism (Figure 1A). Among them, the “inflammatory response pathway” had the lowest *p* value and the highest enrichment factor. The data suggest that long-term intake of LCA can help reduce the body’s inflammatory response (50). The up-regulated differentially expressed genes in the DON-treated group compared to the vehicle group were enriched in KEGG, and the enrichment was mainly concentrated in the MAPK signaling pathway, ubiquitin-mediated proteolysis, AMPK signaling pathway, RNA degradation, IL-17 signaling pathway, p53 signaling pathway, cholesterol metabolism, ferroptosis, and steroid biosynthesis (Figure 1B). Cnetplot illustrated the specific genes associated with these pathways (Figure 2A). Down-regulated differentially expressed genes are centrally enriched in KEGG, including MAPK signaling pathway, Wnt signaling pathway, protein processing endoplasmic reticulum, AMPK signaling pathway, apoptosis, endocrine resistance, biosynthesis of amino acids, PPAR signaling pathway, fatty acid metabolism, amino sugar and nucleotide sugar metabolism (Figure 1C). Cnetplot shows specific genes associated with these pathways (Figure 2B). Further function annotations of transcripts are shown in (Figure 1F). The GO and KEGG pathway enrichment analysis of DEGs reveals that genes are the most enriched (*p* < 0.05) MAPK and IL-17 signaling pathways. As shown in the Venn diagram (Figure 1D), there were (DON vs. vehicle) upregulation genes, but DON vs. DON + LCA; there were DON vs. DON + LCA downregulated genes. In addition, (DON vs. vehicle) downregulation genes, but DON vs. DON + LCA; there are 269 (DON vs. DON + LCA) upregulated genes (Figure 1E). The relationship diagram between genes given by STRING showed that MAPK3, TP53, AKT3, and other genes are closely related. This suggests that these genes in IPI-2I cells may interact with each other under DON treatment, leading to responses such as inflammation and apoptosis (Figure 2C).

### 3.2 DON exposure up-regulates inflammatory pathway genes DUSP5 and TRAF5

**Figure 3:**
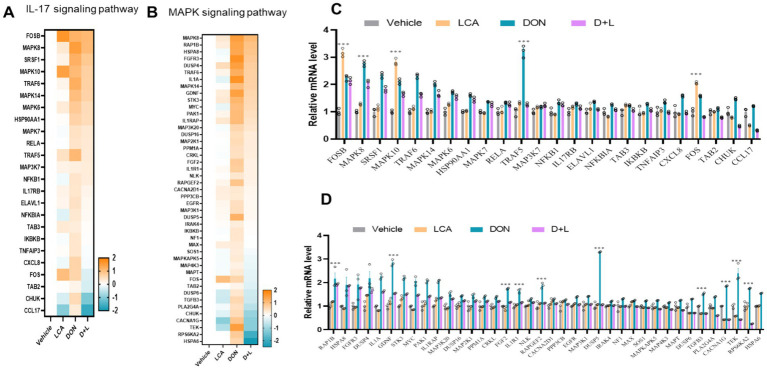
By the KEGG database, the expression levels of genes enriched in IL-17 **(A)** and MAPK **(B)** signaling pathways across various treatment groups were analyzed and compared. Genes with significant changes (Log2 transformed) in expression were identified to generate the heatmaps. qRT-PCR detects the gene expression of key enzymes in IL-17 **(C)** and MAPK **(D)** signaling pathways, the relative expression levels were evaluated by qRT-PCR. *p < 0.05 and ***p < 0.001 compared with the uninfected sample.

As shown in Figures 3A,B, the RNA-seq data set analysis reveals that the genes involved in IL-17 and MAPK signaling pathways are upregulated during DON exposure. DON exposure significantly upregulated (*p* < 0.05) the relative mRNA expression level of MAPK8 and TRAF5 (Figure 3C). An upregulated (*p* < 0.05) relative mRNA expression level of RAP1B, GDNF, FGF2, IL1R1, RAPGEF2, DUSP5, TGFB3, CACNA1G, TEK and RPS6KA2 were noted in IPI-2I exposed to DON (Figure 3D).

### 3.3 Histone modifications are involved in the regulation of LCA-rescued DUSP5 and TRAF5

**Figure 4:**
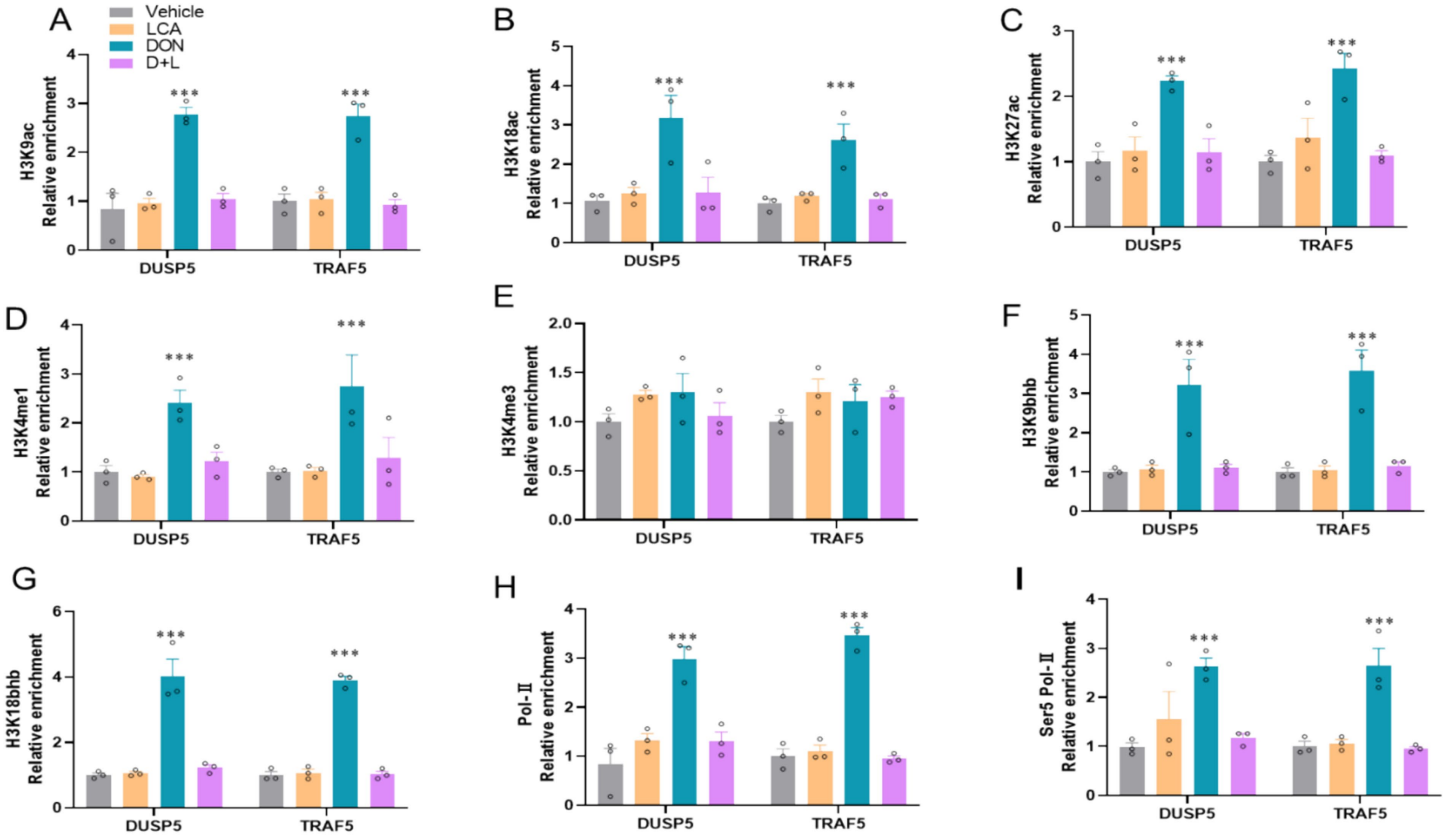
DON exposure modifies histone modification at the locus of DUSP5 and TRAF5. The relative enrichment of histone marks’ **(A)** H3K9ac. **(B)** H3K18ac. **(C)** H3K27ac. **(D)** H3K4me1. **(E)** H3K4me3. **(F)** H3K9bhb. **(G)** H3K18bhb occupancy was analyzed by ChIP–qPCR. **(G,H)** The relative enrichment of co-activator **(H)** RNA polymerase II. **(I)** RNA polymerase II Ser 5 at the locus of DUSP5 and TRAF5. *p < 0.05 and ***p < 0.001 compared with the uninfected sample. The circles represent the distribution of results for different samples.

Since modulations of IL-17 and MAPK signaling pathways are associated with epigenetic regulations, we used ChIP-qPCR to detect histone mark enrichments in DUSP5 and TRAF5 genes. DON-exposed IPI-2I cells dramatically enhanced (*p* < 0.05) histone marks associated with transcriptional activation, H3K9ac, H3K18ac, H3K27ac, H3K4me1, H3K9bhb, and H3K18bhb at the enhancers of DUSP5 and TRAF5, respectively (Figures 4A–G). However, H3K4me3 is not significantly increased. DON-exposed IPI-2I cells significantly increase (*p* < 0.05) the recruitment of the active cofactor RNA polymerase II (Pol-II) and RNA polymerase II serine 5 phosphorylated (Ser5 Pol-II) to target enhancers of DUSP5 and TRAF5 (Figures 4H,I). These findings demonstrate the important roles of cofactors and histone modifications in regulating DUSP5 and TRAF5 in ileum epithelial cells exposed to DON.

## 4 Discussion

The most common mycotoxin is DON, which is widely distributed and commonly found in human food and animal feed. It causes gastrointestinal disorders, systemic immunity, and other diseases (3). Pigs are more sensitive to DON than other animal species. DON can cause immune system problems, diarrhea, vomiting, poor nutrient growth and absorption, and financial loss (51). Exposure to DON causes apoptosis, oxidative stress, cytotoxicity, and intestinal barrier disruption (52). Additionally, it is a ribotoxic mycotoxin that causes inflammation and oxidative stress (53). The gastrointestinal tract is the first line of defense against infections and toxins from the outside world and is essential for cellular and systemic immune responses (54). Several *in vitro* and *in vivo* investigations have shown that DON compromises intestinal barrier integrity and regulates cell proliferation and function, affecting IEC function (55, 56). Ingestion of grains stained with DON can affect animal or human health differently. Despite the extensive literature on the cytotoxic effects of DON, less research has been conducted to mitigate its toxicity. To reduce the harm caused by DON and to improve the intestinal health of animals, it is necessary to develop efficient nutritional and prevention methods. Although LCA is frequently used to promote growth and development in animals due to its anti-inflammatory and liver-protective properties, reports on the protective benefits of LCA in reducing DON-induced intestinal inflammation, apoptosis, and its active mechanism are rare. Therefore, using RNA-seq analysis and molecular biochemical techniques, this study revealed the potential mechanism of LCA for histone modifications in DUSP5 and TRAF5 genes in DON-induced IPI-2I cells. This supports using LCA as a feed additive to reduce DON cytotoxicity in feeding animals.

To investigate the differential gene expression profiles of the different treatment groups in this work, RNA-seq was used to elucidate the induction mechanisms of LCA further to alleviate cell damage caused by DON. The result demonstrated that the level of DEGs was different when comparing two groups. GO and KEGG enrichment analyses of DEGs were performed to examine the role of genes. Immune and inflammatory responses were found to be important factors affecting DEGs. IL-17 and MAPK signaling pathways are critical for the inflammatory response. The intestinal epithelial barrier can be disrupted by directly promoting or inhibiting intestinal cell growth, permeability, and death via IL-17 and MAPK signaling pathways (57, 58). Besides pro-inflammatory variables, DON can increase several IL-17 and MAPK pathways. The current study identified the differentially expressed IL-17 and MAPK signaling pathways, such as IL1RAP, MAPK14, STK3, DUSP16, DUSP5, and TRAF5 in the DON vs. vehicle. DUSP5 and TRAF5 are key proinflammatory factors such as TNF-α, IL-6, and IL-1β (40, 44); it is formed by various cell types such as platelets, fibroblasts, epithelial cells, and CD4+ T lymphocytes (45). DON can activate the NF-κB signaling pathway, activating MAPK signaling pathways. These signaling pathways are important for regulating inflammation by facilitating the synthesis of inflammatory proteins (59). Recent research comparing transcriptomes in IPI-2I cells with and without DON administration showed that activation of the p38 MAPK and Erk1/2 pathway results in inflammation (3). Although this study found enrichment in the same MAPK, TNF, NF-κB signaling pathways, and cytokine-cytokine receptor interaction, there were not many common DEGs between the two studies when using the same DEG detection threshold. Common DEGs were detected including MAP3K5, CXCL8, IL1A, IL-6, FOS and IL1. Of these, only eight genes, including FOS and IL1A, exhibited variable expression levels, confirming the validity of the findings in both investigations. These results demonstrate the involvement of chemokines, TNF, MAPK, and NF-κB signaling pathways in the inflammatory and immune response of DON-stimulated IPI-2I cells.

In the present study, we observed that DON exposure significantly upregulated the IL-17 signaling pathways of gene MAPK8 and TRAF5 and MAPK signaling pathways of gene RAP1B, GDNF, FGF2, IL1R1, RAPGEF2, DUSP5, TGFB3, CACNA1G, TEK and RPS6KA2 in the IPI-2I. TRAF5 helps activate the NF-κB pathway, an essential mechanism for the transcription of genes linked to immune responses and inflammation (60). TRAF5 may activate the MAPK pathway that regulates gene expression, cell division, and proliferation (61). Furthermore, DUSP5 over-expression results in long-term inflammation through NF-κB activation in irradiated human arteries (62). The ability of DUSP5 to bind and inactivate ERK1 and ERK2 *in vivo* is highly specific (63). Previous studies have demonstrated that DUSP5 is localized in the nucleus and regulates nuclear ERK activation (64). As obesity develops, DUSP5 mRNA expression rises with an increase in TNFα expression (42). It was demonstrated that MAPK-specific DUSPs act as essential downstream regulators of MAPK activation and inflammation (65).

It is well known that LCA, a secondary bile acid, can affect gene expression by modifying histones and other epigenetic modifications. The participation of LCA in histone modifications in DUSP5 and TRAF5 genes in ileum epithelial cells reveals a complex regulatory mechanism in the context of DON exposure, a mycotoxin affecting intestinal cells (3, 66). NF-κB and MAPK signaling pathways, which play important roles in inflammatory responses, are activated by TRAF5-mediated signal transduction from TNF receptors. Expression levels of TRAF5 may be affected by LCA-induced histone modifications, which may further alter immune responses and inflammation (66, 67). Transcription factors, meanwhile, bring enzymes into DNA; these enzymes typically act by acetylation and acetylation histone tails (68). We observed that DON-exposure significantly increased H3K9ac, H3K18ac, H3K27ac, H3K4me1, H3K9bhb, and H3K18bhb enrichments on DUSP5 and TRAF5 in IPI-2I cell as well as RNA Pol-II and Ser5 Pol-II. If LCA-induced histone modifications decrease the expression of DUSP5 and TRAF5, it may upregulate the MAPK pathway, which may reduce inflammatory signals such as NF-κB and provide protection against damage caused by DON.

In IPI-2I cells, DON exposure causes inflammation and apoptosis; these effects can be reduced by adding LCA. MAPK and IL-17 signaling pathways are key players in DON-induced inflammation and apoptosis. However, by upregulating these and other inflammatory and immune-related gene expressions, LCA exerts a mitigating effect via the MAPK and IL-17 signaling pathway (Figure 3). In summary, this study reveals that an increase in histone modification is involved in DON-induced IPI-2I cells by modulating the transcriptional inhibition of the DUSP5 and TRAF5 genes. LCA could act against DON-induced IPI-2I cell damage, thereby alleviating histopathological lesions by rescuing the histone modification-dependent transcriptional activation of the DUSP5 and TRAF5 genes. This work provides new insights into the epigenetic mechanism of LCA in porcine intestinal epithelial cells against damage caused by mycotoxin exposure. This establishes a theoretical basis for developing and using LCA as a feed additive to mitigate DON-induced cytotoxicity and improve animal health and productivity.

## Data Availability

The original transcriptome data proposed in this study has been preserved at the National Center for Biotechnology Information (https://www.ncbi.nlm.nih.gov/), and the preservation number is PRJNA1095391.

## Footnotes

^1^ http://metascape.org/

^2^ https://david.ncifcrf.gov/

^3^ http://www.bioinformatics.com.cn

^4^ https://cn.string-db.org/
